# When Cooperation Was Efficient or Inefficient. Functional Near-Infrared Spectroscopy Evidence

**DOI:** 10.3389/fnsys.2017.00026

**Published:** 2017-05-09

**Authors:** Michela Balconi, Maria E. Vanutelli

**Affiliations:** ^1^Research Unit in Affective and Social Neuroscience, Catholic University of MilanMilan, Italy; ^2^Department of Psychology, Catholic University of MilanMilan, Italy

**Keywords:** cooperation, feedback, self-efficacy, interpersonal strategies, emotions, fNIRS

## Abstract

Cooperation is a construct within social cognition that requires both self-perception and the comprehension of others' actions. In the case of synchronized activities the adoption of common strategies is crucial, but this process can be strongly influenced by those variables. In fact, self-perceived efficacy within the social exchange can affect the motivational components toward the creation of synergic actions. Thus, what happens when our performance is efficient or inefficient during cooperation? This question was answered in the present study where we compared behavioral performance and neural activation across different conditions where subjects received an external feedback assessing a good or a poor outcome during a cooperative game. The request was to synchronize responses in a way to achieve good cooperation scorings. Results showed that the behavioral performance was affected by feedback valence, since the negative feedback induced a significant worse performance in contrast to the positive one, which significantly increased performance. For what concerns neural activation, data from functional near-infrared spectroscopy (fNIRS) showed a specific lateralization effect with the right DLPFC recruited in the case of negative feedback, and an opposite left-sided effect in the case of a positive feedback. Findings were interpreted by proposing that the inefficient condition could be similar to a competitive context since the perception of a failed joint action could have frustrated the cooperative attitude and the use of joint strategies.

## Introduction

When we speak about cooperation we usually refer to collaborative inter-actions that occur between two or more actors with the aim to pursue common goals. This kind of behavior is addressed toward the realization of a definite objective that can provide benefits to all the people involved. While chasing such targets, a set of cognitive and affective mechanisms arise and support behavior (Balconi et al., 2012; Balconi and Canavesio, 2013, 2014; Liu et al., 2015, 2016; Balconi and Vanutelli, 2016a; Vanutelli et al., 2016). Moreover, during cooperation, the behavioral performance essentially entails the involvement of social cognition processes (Decety et al., 2004; Declerck et al., 2010). For example, previous research explored the effects of cooperation in relation to self-perception, self-efficacy, and social cognition within social interactive contexts. Findings revealed that a cooperative condition may reinforce group membership, social cohesion, self-efficacy and the perception of a high rank within a social hierarchy (Goldman et al., 1977; Funane et al., 2011; Cui et al., 2013; Balconi and Pagani, 2015; Chung et al., 2015). According to recent studies on brain functioning, social cognition and self-efficacy are represented within prefrontal areas (Iacoboni et al., 2004; Mitchell et al., 2005a,b; D'Argembeau et al., 2007; Beer et al., 2009; Wagner et al., 2012; Jenkins et al., 2014). Indeed, it was observed that the neural circuits linking the limbic system and the prefrontal cortex (PFC) may support the emotional and cognitive components of social interactions during cooperation (Levitan et al., 2000). Specifically, it was found the Dorsalateral Prefrontal Cortex (DLPFC), the Orbitofrontal Cortex (OFC) and some portion of the Frontal Gyrus (FG) are generally recruited during interpersonal cooperation (Chiao et al., 2009; Balconi and Pagani, 2014, 2015; Wang et al., 2016). Strong inter-brain neural synchrony was observed in the posterior region of the right middle (MFG) and superior frontal gyrus (SFG) during cooperative joint actions, suggesting that this area could be involved in goal-oriented social interaction such as complex dynamics and social decision-making (Baker et al., 2016; Liu et al., 2016). The same patterns of inter-brain synchrony during cooperation was also observed in the dorsomedial prefrontal cortex (dmPFC).

The activation of prefrontal regions during social interactions that implicate a cooperative action probably reflects the recruitment of brain networks that can provide top–down control over cognitive processes and emotional responses involved during cooperative joint interactions in a way to prepare the best behavioral response (Marsh et al., 2009). Indeed, these brain areas are usually recruited during the regulation of cognitive and socio-emotional responses (Gray et al., 2002; Ochsner and Gross, 2005; Knyazev, 2007; Ray and Zald, 2012). Nonetheless, as emerged in many previous studies, a psychological construct has been considered to mediate the neural response during the participation in cooperative joint-actions, that is the evaluation of the behavioral outcome as efficient or inefficient. In fact, this perception is a powerful reinforce that can both influence goal-directed behaviors and train the brain to work jointly. Previous experiment already investigated the effects of positive outcomes on self-perception, behavioral performance and neural correlates for cooperative or competitive tasks based on the effects of an external feedback (Monterosso et al., 2002; Balconi and Vanutelli, 2016a,b). Results showed that the perception of positive feedbacks is able to induce a positive behavioral performance and is related to the activation of prefrontal sites. In fact, it is associated with an increase in cognitive synergy and brain-to-brain coupling (Baker et al., 2016). Nevertheless, although the role of an external feedback was considered as a key factor in cooperative tasks, no previous study directly compared the influence of a positive vs. a negative feedback on performance and neural correlates. In detail, what kind of neural and behavioral response can we observe when we observe to be efficient or, on the contrary, inefficient during the adoption of joint strategies?

Therefore, it is relevant and urgent to distinguish the perception of our social efficacy or inefficacy and their effects in different interpersonal conditions—namely in cooperative situations with a negative feedback or with a positive feedback, which are qualitatively distinct psychological domains. Different possible ways are suggested to differentiate unsuccessful from successful forms of cooperation. Firstly, from a cognitive point of view, we may suppose that in the case of failure perception a higher mental effort is required to represent a sort of “dysfunctional” interaction where the synergic strategy is disrupted. Indeed, based on the interaction modalities (positive or negative cooperation), individuals may either facilitate or obstruct others' goal achievement (such as in competition). Concurrently, they can self-represent themselves as less proficient in relation to the common task. Supplementary research demonstrated that one's own actions are facilitated when others' actions are at the disposal of common interests (as in cooperation compared with competition) and when they are efficient (as in successful cooperation; Knoblich and Jordan, 2003; Sebanz et al., 2003). In contrast, in the case of inefficient cooperation, the interlocutors' behavior and the outcomes of the joint-action are less predictable than in the case of effective cooperation, where there is a planned expectation for the other agent's behavior and the positive outcomes. As found for competitive tasks, it is plausible that we need to determine agents' mental state that is decoupled from reality, and to handle simultaneously these two competing views (the real one about inefficacy and the desirable one for a good performance) on the interactions (Leslie, 1987; Gallagher and Frith, 2003). As such, this condition may impose an increase in cognitive load, due the selection of salient information or response to achieve a new goal internally represented (Humphrey, 1988; Nigg, 2001). Thus, the strong increase in the prefrontal activity—mainly the mPFC—observed during competition or in the case of a failure may in part mirror higher executive processing demands (Decety et al., 2004). Specifically, it was shown that the processing load associated with competitive conditions results in heightened cortical activity across these brain regions. Similarly, an unsuccessful strategy, although in a cooperative context, may require an increased demand of cognitive reserves to update and change the old strategy in favor of a new direction.

Secondly, we may suggest that an inefficient compared with an efficient cooperation may induce more negative emotions with a concomitant withdrawal behavior toward their own partner due the frustrated joint-action. That is, an increased perception of inefficacy based on joint-strategies may induce a decreasing in the positive effect produced by the cooperative task, with a sense of impotence and failure. A further hypothesis deals with the necessity of a sort of reparative strategy, to compensate the reciprocal inefficacy and to try to reach a more proficient common strategy. Here, the second option may be represented as a sort of a resume in order to strengthen the common goals and obtain a better result. In this second case we can assume a sort of renewed cooperation to compensate a previous failure. This should involve more frontal cortical portions related to prosocial support and emotional empathic response, as reported in some studies (Balconi and Canavesio, 2013a,b, 2014). In addition, based on the valence model of emotions, a lateralized responsiveness should suggest a right-sided than a left-sided prefrontal activity (Demaree, 2005; Balconi et al., 2009, 2015a,b; Harmon-Jones et al., 2010). Indeed the emotional negative impact of an inefficacy-related feedback should induce significant lateralized PFC activation as suggested by the valence and the approach-avoidance model of emotions. At this regard, we may consider the increased convergence in the right hemisphere as a possible marker of non-achieved goals. Indeed, as previously observed, the frontal cortical asymmetry in favor of the right hemisphere is associated with withdrawal motivation in opposition to approach motivation (Davidson, 1993; Jackson et al., 2003; Urry et al., 2004; Balconi and Mazza, 2010; Harmon-Jones et al., 2010; Koslow et al., 2013). Therefore, in the present study we explored the cortical response to two opposite conditions based on feedback.

Due to their fast temporal evolution, social processes during interactive contexts should preferably be explored by means of imaging methods that can provide good resolution in both temporal and spatial components, that are more ecologically valid and usable in dynamic and interactive conditions. For these reasons we used event-related hemodynamic responses thanks to functional Near-Infrared Spectroscopy (fNIRS; Elwell et al., 1993). Therefore, we implemented a specific paradigm which monitored the feedback (for efficient or inefficient performance) just before (four time intervals) and just after (four time intervals) the positive/negative feedback. Based on previous hypotheses the feedback (artificially manipulated) should ingenerate different PFC responsiveness. Indeed an increased DLPFC activity was attended in the case of a negative feedback, mainly right lateralized due to negative emotions and to a more unpredictable outcome form the relational point of view. As found in previous research, a specific increased prefrontal activity is attended in order to manage an unexpected and more complex situation (failure), since subjects could realize to be inefficient in joining their strategies and actions. In addition, the increased work load for the unattended negative outcome should induce a worse performance in post-feedback, with significant increasing of response time (RTs) and error rates (ERs). In contrast, the left DLPFC is expected to be more responsive in the case of a positive feedback, with a concomitant better cognitive performance induced by a potentiated sense of self-efficacy and by the social reinforce.

## Materials and methods

### Participants

Fifty undergraduate students (*M* = 23.13, *SD* = 2.32; male = 23) took part in the experiment. The participants were all right-handed and presented normal or corrected-to-normal visual acuity. Exclusion criteria were history of psychopathology (Beck Depression Inventory, BDI-II, Beck et al., 1996) for the subjects and immediate family. Also State-Trait-Anxiety-Inventory (STAI, Spielberger et al., 1970) was submitted before the experimental session. Based on the psychometric measures and the clinical screening, no neurological or psychiatric pathologies were observed. Subjects gave informed written consent to participate in the study and no payment was provided for their participation. Finally, the research was approved by the local ethics committee of the Department of Psychology, Catholic University of Milan.

### Procedure

Subjects performed a simple task for sustained selective attention (it was a modified version of (Balconi and Vanutelli, 2016a). They were seated in a half-darkened room in front of a PC screen CRT positioned nearby 60 cm in front of their eyes (refresh 60 Hz). They were seated side-by-side, but separated by a black screen to prevent visual contact.

**Figure 1:**
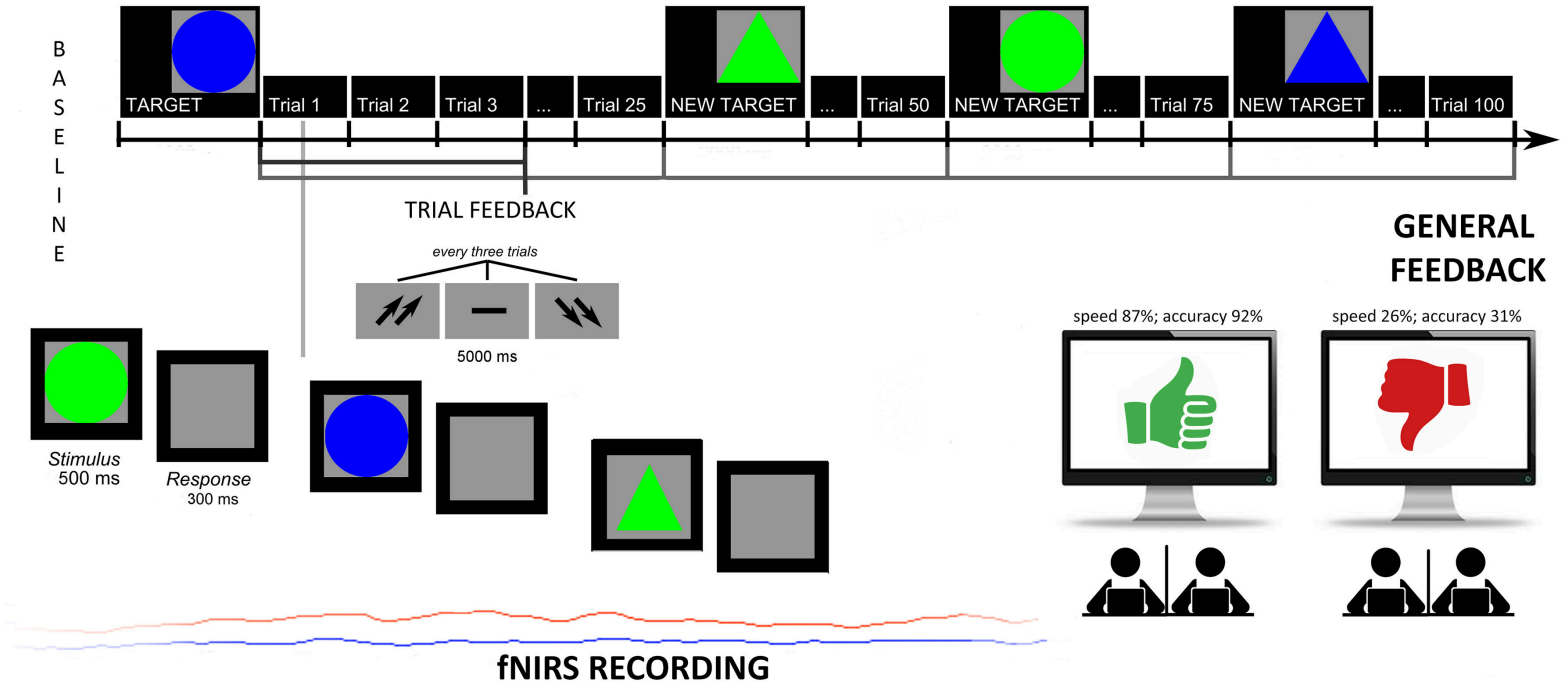
**Experimental task**. Experimental procedure which represents the setting, the experimental tasks and fNIRS recording.

Subjects were told that their performance would have used to evaluate the subjective skills and, to strengthen their motivation, that these measures are usually applied to screen individuals' future professional success and teamwork capabilities. In addition, the cooperative nature of the task was stressed since they were told to synchronize their responses, in term of accuracy (number of correct responses) and response times (RTs) with a second agent to obtain better outcomes. The task consisted in recognizing target figures from non-targets: after three figures (a trial) subjects received a feedback signaled by two up-arrows (good cooperation outcomes); a dash (mean performance); or two down-arrows (scarce cooperation outcomes). They were requested to answer by using a mouse. The modified version of the task was composed by two sessions: the first which did not include a specific feedback to performance (4 blocks before the feedback, 100 trials); the second which included a specific negative feedback to the performance (4 blocks with the feedback, 100 trials). Halfway, in fact, participants received a general evaluation of their cooperative performance, either positive or negative based on the two different conditions. Both the feedbacks and the evaluation were fixed, and subjects were told they had a good cooperation (score with 87% in terms of speed synchrony, and 92% in terms of accuracy synchrony); or a bad cooperation (score with 26% in terms of speed synchrony, and 31% in terms of accuracy synchrony). They were also encouraged to keep (in the case of positive feedback) or modify (in the case of negative feedback) their performance level during the second part of the experiment. Across the task, after an initial mean performance, subjects were constantly informed about their cooperation by presenting the up-arrows or down-arrows in 70% of cases, while the other 30% of cases the two other possibilities (see Figure 1).

Based on a post-experimental questionnaire, participants reported they were strongly engaged in the cooperative context (94% told to be strongly engaged), to trust the feedback about the performance (96%), and to consider the task as relevant for their social status (93%). About their perception of self-efficacy, the two groups were requested to consider their efficacy (on Likert scale 1–7 points, for group 1 *M* = 6.45, *SD* = 0.12; for group 2 *M* = 2.76, *SD* = 0.17). Significant differences were found between the groups [*F*_(1, 49)_ = 7.23, *p* ≤ 0.001, η^2^ = 0.32].

### Performance scoring

The response times (RTs, ms) were recorded from the stimulus onset, and the error rates (ERs) were calculated as the total number of incorrect detections out of the total trial. Therefore, higher values represented increased incorrect responses.

### fNIRS

**Figure 2:**
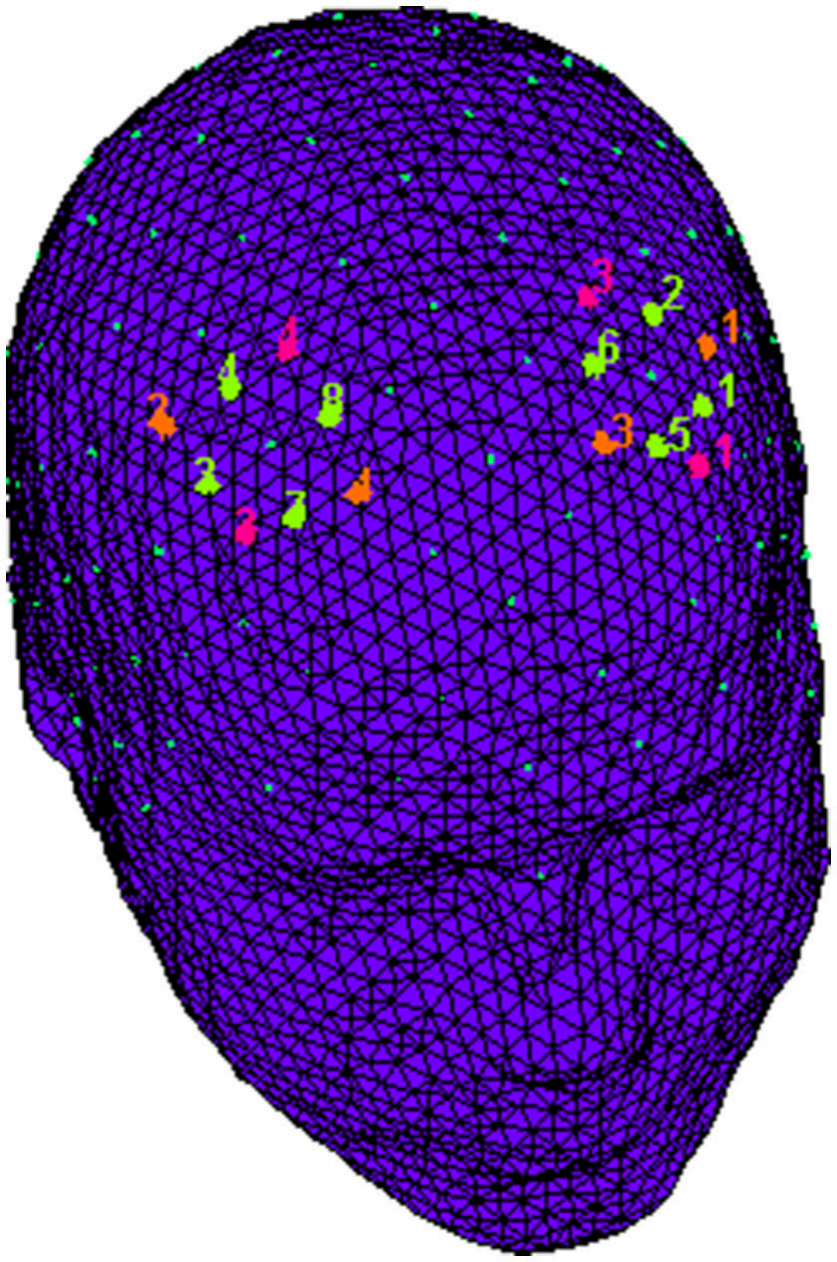
**fNIRS montage**. The location of NIRS channels. The emitters (orange) were placed on positions FC3-FC4 and F1-F2, while detectors (fuchsia) were placed on FC1-FC2 and F3-F4. Resulting channels (green) were as follows: Ch 1 and Ch 3 correspond to the left and right DLPFC. Ch 2 and Ch 4 correspond to the left and right PMC. Ch 5 and Ch 7 correspond to the left and right FEF. Ch 6 and Ch 8 correspond to the left and right SFG.

fNIRS recordings were conducted with NIRScout System (NIRx Medical Technologies, LLC. Los Angeles, California) using a 8-channel array of optodes (4 light sources/emitters and 4 detectors) covering the prefrontal area. Emmiters were placed on positions (FC3-FC4 and F1-F2) while detectors were placed on FC1-FC2 and F3-F4; Figure 2. Emitter-detector distance was kept at 30 mm for contiguous optodes and a near-infrared light of two wavelengths (760 and 850 nm) was used. NIRS optodes were placed on the subject's head by using a NIRS-EEG compatible cup according to the international 10/5 system. Resulting channels are reported: Ch 1 (FC3-F3) and Ch 3 (FC4-F4) correspond to the left and right (respectively) DLPFC (Brodmann Area 9). Ch 2 (FC3-FC1) and Ch 4 (FC4-FC2) correspond to the left and right (respectively) Premotor Cortex (PMC, Brodmann Area 6). Ch 5 (F1-F3) and Ch 7 (F2-F4) correspond to the left and right (respectively) Frontal Eye Fields (FEF, Brodmann Area 8). Ch 6 (F1-FC1) and Ch 8 (F2-FC2) correspond to the left and right (respectively) (SFG, Brodmann Area 6; Koessler et al., 2009). With NIRStar Acquisition Software, changes in the concentration of oxygenated (O2Hb) and deoxygenated hemoglobin (HHb) were acquired from a 120 s resting baseline. Signals recorded from the 8 NIRS channels were acquired with a sampling rate of 6.25 Hz and analyzed and transformed in values for the changes in the concentration of oxygenated and deoxygenated hemoglobin, for each channel, scaled in mmol*mm. O2Hb and HHb changes were calculated by using the optical density changes of 760- and 850-nm lights in accordance to the modified Beer-Lambert law. The raw data from each channel were digitally band-pass filtered at 0.01–0.3 Hz. Then, the mean concentration of each channel was calculated by averaging data across the trials, starting from the trial onset for the following 5 s. According to the mean concentrations in the time series, the effect size in every condition was calculated for each channel and subject. The effect sizes (Cohen's d) were computed as the difference of the mean of the baseline and trial divided by the standard deviation (SD) of the baseline: *d* = (m1–m2)/s, with m1 and m2 being the mean concentration values during baseline and trial, respectively, and s the SD of the baseline. In this case, the baseline was calculated considering the 5 s immediately before the trial. Then, the effect sizes obtained from the 8 channels were averaged in order to increase the signal-to-noise ratio. Although NIRS raw data were originally relative values and could not be directly averaged across subjects or channels, effect sizes normalized data could be averaged regardless of the unit (Schroeter et al., 2003; Matsuda and Hiraki, 2006; Shimada and Hiraki, 2006). In fact, the effect size is not affected by differential pathlength factor (DPF; Schroeter et al., 2003).

## Results

Three sets of analyses were performed with respect to behavioral (ERs; RTs) and neurophysiological (fNIRS: O2Hb measures) measures.

A preliminary repeated measure ANOVA with independent factor Condition (Cond: pre vs. post feedback) and Feedback (Fed; positive vs. negative) was applied to ERs and RTs. In the case of neurophysiological measure (O2Hb) for the ANOVA two repeated factors were added to Cond and Fed, that is localization (Loc: DLPFC, SFG, PMC, FEF) and lateralization (Lat: left vs. right) independent factor. For all of the ANOVA tests, the degrees of freedom were corrected using Greenhouse–Geisser epsilon where appropriate. Post-hoc comparisons (contrast analyses) were applied to the data. Bonferroni test was applied for multiple comparisons. In addition, the normality of the data distribution was preliminary tested (tests for kurtosis and asymmetry were applied). The normality assumption of the distribution was supported by these preliminary tests.

To exclude a possible learning effect, a further analysis was applied, comparing separately the first set of four intervals (before feedback) and the second set of four intervals (post feedback) for all the dependent measures (RTs, ERs, O2Hb). Since no significant differences among the four intervals before and after the feedback were found for both the positive/negative feedback condition, we did not include this factor in the successive analysis.

### RTs and ERs

**Figure 3:**
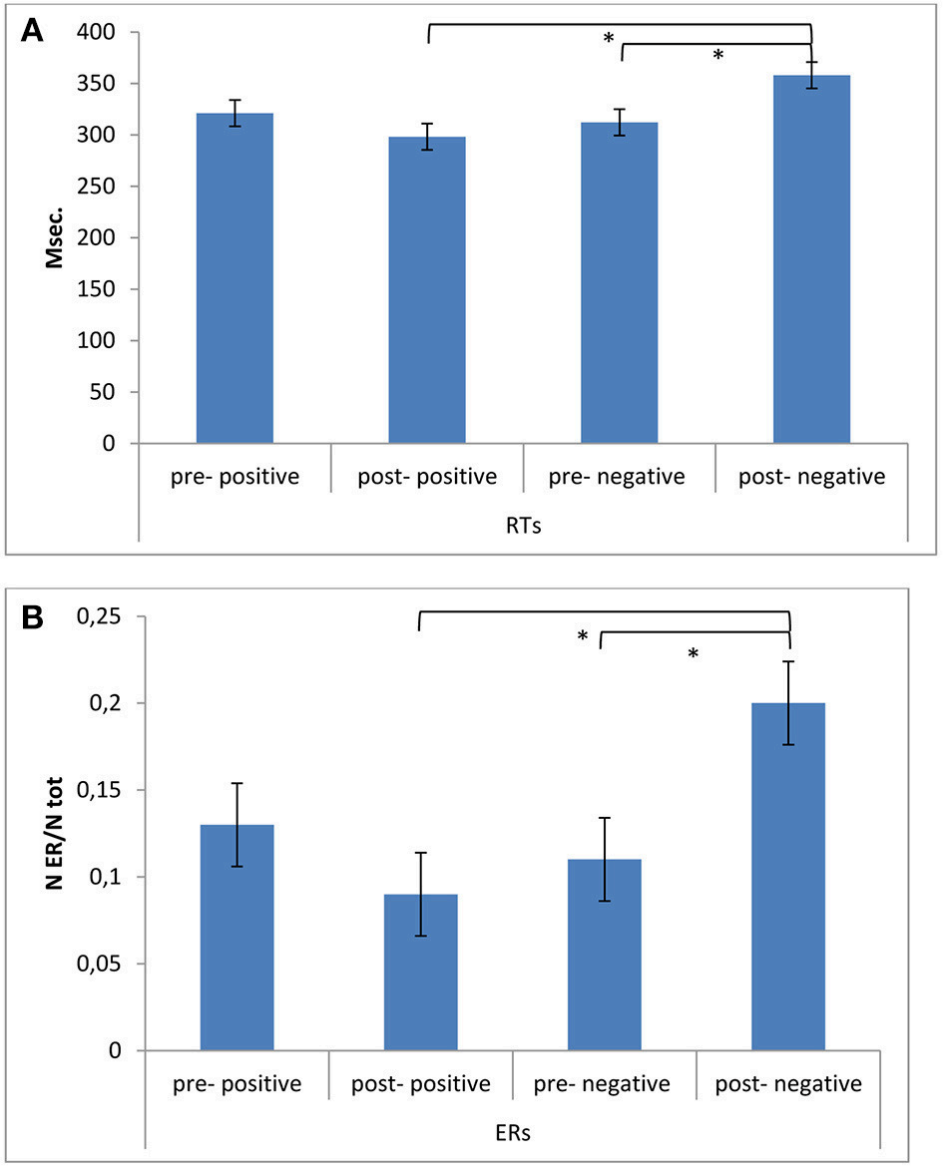
**Behavioral results. (A)** RTs modulation as a function of Condition (pre vs. post) and Feedback (positive vs. negative). The speed performance was characterized by longer RTs during post-feedback condition when a negative reinforce was provided. **(B)** ERs modulation as a function of Condition (pre vs. post) and Feedback (positive vs. negative). Accuracy performance was characterized by decreased performance (higher ERs) during post-feedback condition in the case of a negative feedback. ^*^ ≤ 0.01.

As shown by the ANOVA, significant differences in ERs were found for Cond × Fed [*F*_(1, 49)_ = 11.07, *p* ≤ 0.001, η^2^ = 0.43] (Figures 3A,B). Indeed, a decreased performance (higher ERs) was found for post-feedback condition compared to pre-feedback in the case of a negative feedback [*F*_(1, 49)_ = 11.07, *p* ≤ 0.001, η^2^ = 0.43]. In addition ERs were higher in post-feedback condition in the case of a negative than a positive feedback [*F*_(1, 49)_ = 9.13, *p* ≤ 0.001, η^2^ = 0.40]. No other *post-hoc* comparison was significant.

About RTs, a significant effect was found for Cond × Fed [*F*_(1, 49)_ = 7.71, *p* ≤ 0.001, η^2^ = 0.34], with increased RTs in post-feedback than pre-feedback condition in the case of a negative feedback [*F*_(1, 49)_ = 8.77, *p* ≤ 0.001, η^2^ = 0.39]. In addition RTs were increased in post-feedback condition in the case of a negative than a positive feedback [*F*_(1, 49)_ = 9.50, *p* ≤ 0.001, η^2^ = 0.40]. No other *post-hoc* comparison was significant.

### fNIRS

**Figure 4:**
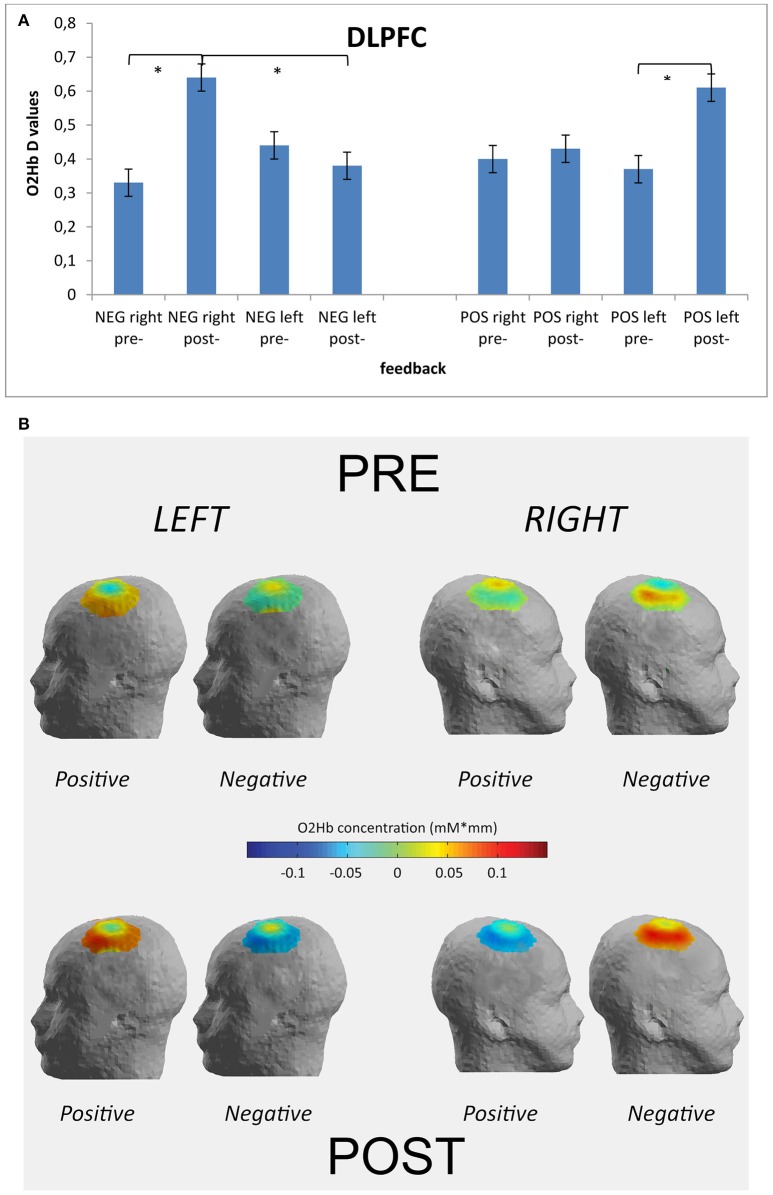
**Optical imaging (fNIRS) results**. Histograms **(A)** and activation maps **(B)** of O2Hb variations (*D*-values) for Condition × Feedback × Localization × Lateralization. The post-feedback condition was characterized by increased *D*-values over the right DLPFC after the negative feedback and over the left DLPFC after the positive feedback. ^*^ ≤ 0.01.

The analysis on HHb did not reveal significant effects, and for this reason we reported only results for O2Hb-values. Indeed, the main and interaction effects did not reveal relevant differences based on the independent factors. This effect may be due to the fact that the increase in O2Hb is larger than the decrease in HHb. Repeated measure ANOVA showed significant effect for Cond × Fed [*F*_(1, 49)_ = 8.77, *p* ≤ 0.001, η^2^ = 0.37] and Cond × Fed × Lat × Loc [*F*_(1, 148)_ = 9.41, *p* ≤ 0.001, η^2^ = 0.39]. Indeed, a general increased activity was found in post-feedback condition than in pre-feedback in the case of negative feedback [*F*_(1, 49)_ = 8.30, *p* ≤ 0.001, η^2^ = 0.37]. Secondly, as shown by contrast analyses applied to the simple effects for the second significant interaction, the right DLPFC activity was higher in post-feedback condition more for negative than for positive feedback [*F*_(1, 49)_ = 8.23, *p* ≤ 0.001, η^2^ = 0.35]. In addition in the case of negative feedback right DLPFC activity in post-feedback condition was increased than right DLPFC in pre-feedback condition [*F*_(1, 49)_ = 7.11, *p* ≤ 0.001, η^2^ = 0.35]. Finally, the left DLPFC activity in post-feedback condition was increased than left DLPFC in pre-feedback condition in the case of positive feedback [*F*_(1, 49)_ = 9.40, *p* ≤ 0.001, η^2^ = 0.40] (Figures 4A,B). No other effect was statistically significant.

### Correlational analysis

**Figure 5:**
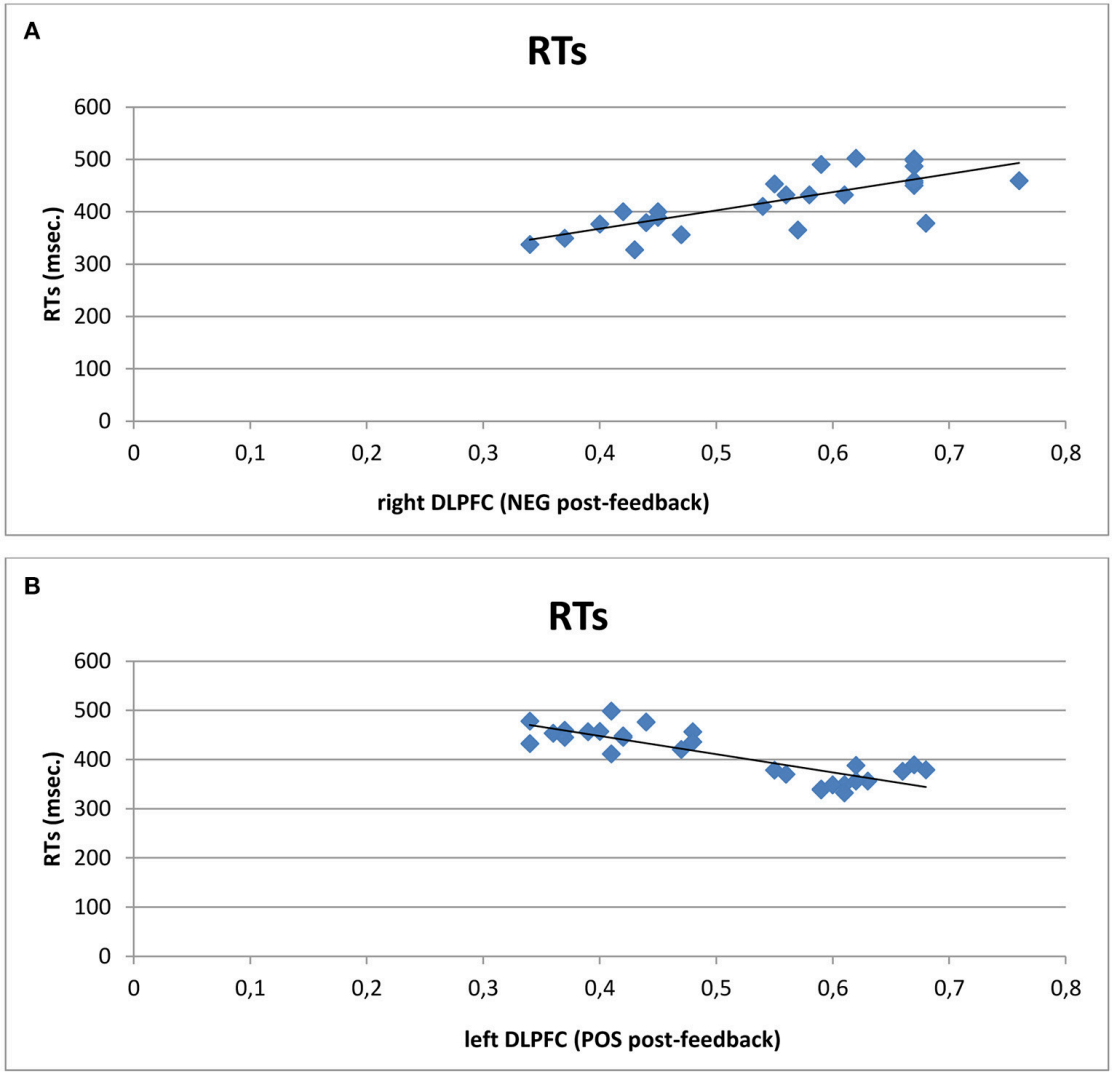
**Correlational analyses**. Scatterplots displaying Pearson's coefficients between right **(A)** and left **(B)** DLPFC with RTs. Results showed that increased right DLPFC activity was positively correlated with the increased RTs values in post-feedback condition. Similarly, RTs decreasing was significantly correlated with left DLPFC activity in the case of positive feedback.

A series of correlation analyses was applied to cognitive performance (ERs; RTs) and O2Hb modulation for each cortical area within the left and right hemisphere, distinctly for each condition (pre- and post-feedback) and each feedback type (positive vs. negative). Pearson correlation coefficients were calculated. We reported only significant correlational results. RTs revealed significant positive correlation with right DLPFC activity in post-feedback condition for negative feedback (*r*^2^ = 0.523, *p* ≤ 0.001): the increased right DLPFC was positively correlated with the increased RTs values in post-feedback condition. Similarly, RTs decreasing was significantly correlated with DLPFC increased activity within the left side in the case of positive feedback (*r*^2^ = −0.543, *p* ≤ 0.001; Figures 5A,B).

## Discussion

The present research explored the effects of efficient vs. inefficient joint-actions in a cooperative task. Indeed the effect of a positive (artificially induced sense of efficacy) or a negative (artificially induced sense of inefficacy) feedback was considered, considering both brain responsiveness and behavioral performance. Specifically, brain activation was acquired during a cooperative task which ingenerates a positive vs. a negative outcome. The following main results were observed. A first main effect was related to the systematic impact of the feedback on the frontal sites, mainly for some specific prefrontal regions (DLPFC). Secondly, a specific lateralization effect was detected, in relation to the nature of the feedback. Indeed, the DLPFC showed a significant higher right activity when the feedback was negative, whereas an increased left DLPFC activity was revealed in the case of a positive feedback. Thirdly the cognitive performance was affected by the feedback, since the negative feedback induced a significant worse performance in contrast to the positive feedback which induced a significant improved performance.

The first main effect was related to the general increased PFC activity after the subjects received their feedback in comparison to the absence of a feedback. This effect was mainly distributed within a specific area that is the DLPFC, whereas other areas were not implicated by feedback processing. This result may be coherent with the suggested hypothesis that the feedback on subjects' performance has a significant impact on their brain activity. In fact, as shown by previous research, the DLPFC may support our social cooperative or competitive interactions with relevant modulations of the brain responsiveness (Rilling et al., 2002, 2007; Nihonsugi et al., 2015; Balconi and Vanutelli, 2016a). As previously shown, the DLPFC has a specific and crucial role in social contexts, in the self-perception of social hierarchy and in monitoring within of social task (Balconi and Pagani, 2015; Wang et al., 2016). In addition, previous results revealed that prefrontal areas are prominent and relevant in social status regulation and joint actions (Karafin et al., 2004; Haruno and Kawato, 2009; De Vico Fallani et al., 2010; Suzuki et al., 2011). This prefrontal brain area is supposed to have an evolutionary relevance in social perception especially when hierarchy across species and human social groups is significant. Therefore, it is plausible to suppose that this area has specialized mechanisms to perceive and regulate joint-actions.

However, a relevant result was related to the lateralized effect we found, that is the specific right lateralization systematically observed after the negative feedback. A possible interpretation is that this increased activation is due to higher cognitive efforts and processing load associated with the representation of a negative condition, resulted in heightened cortical activity (Gallagher and Frith, 2003; Decety et al., 2004). An unsuccessful strategy, although in a cooperative context, may require an increased demand for cognitive resources to update and modify the joint-action style. As such, this condition may require an increase in cognitive load related to the necessity to recalibrate joint strategies, to implement a more efficient cognitive plan and to include new behavioral directions. We may explain these findings also taking into account some previous results related to competition, where we found that the PFC was mainly activated within the right side in the case of a competitive task (Decety et al., 2004; Balconi and Vanutelli, 2016b). Therefore, the present results seem to suggest that the negative cooperative condition is more similar to a competitive task and that this fact may be due to the increasing difficulty in creating a common mental strategy based on the increased work load.

Nevertheless, a second explanation of the present result, an emotional one, may bring the increased right responsiveness back to a significant prevalence of more negative emotions and avoidance attitudes toward the interlocutor, linked to the inefficient inter-action. It should be ascribed to the negative emotional condition that a frustrating feedback may create. In fact it was observed that the right hemisphere supports aversive situations where individuals have to regulate the conflictual and also divergent goals (Balconi et al., 2012). Therefore, a sort of “negative echo” may be intrinsically related to failure, with a significant increasing of withdrawal attitudes (Davidson, 1993; Jackson et al., 2003; Urry et al., 2004; Balconi and Mazza, 2010; Harmon-Jones et al., 2010; Koslow et al., 2013). In other terms, the social relevance of the task and the inefficient cooperative condition may explain this result, together with the decreased DLPFC response based on the “reduced” cooperation induced by a failed joint-behavior. Subsequently, activity patterns in the frontal cortices can be regarded to be crucially involved in processing emotional conditions which characterize the negative context. Nevertheless, it has to be noted that few studies have tried to connect emotional effects of failing cooperation, taking into account the impact of the emotional behavior on cortical system when it responds to specific social situations. Therefore, future research should better explain the role of emotions and negative feedback to disambiguate their reciprocal relation.

However, a more complete explanation should take into consideration the second lateralization effect, that is the left-sided effect we concomitantly found in the case of a positive feedback. This interesting effect, integrated with the right-negative effect, may definitely argue in favor of an emotional explanation more than an increased workload. Indeed this specific lateralization (negative-right; positive-left) may be hardly explained based on the cognitive effort perspective. In contrast, considering the valence model and the withdrawal-approach perspective on emotions, it may more clearly justify the increased DLPFC responsiveness as a function of the feedback nature and its effect on the emotional behavior.

The present interpretation is also supported by the behavioral results and the significant worse performance (increased RTs and ERs) after the negative feedback, whereas a better cognitive performance (decreased RTs) was revealed in the case of a positive self-represented outcome. Although a cognitive effort due to the task after the feedback may not be excluded a priori, we may suppose that the decreased performance in the post-feedback condition may be due to the negative self-perception and the representation of an inefficient interaction. This fact is supported by both higher RTs and increased ERs, which point out the negative feedback condition as the absolute worst cognitive factor during cooperation. Moreover, the presence of an opposite finding in response to a positive feedback (an increased performance) argues in favor of the emotional impact of the social feedback. In fact this last result could be hardly explained only considering a sort of learning effect (which could justify a better performance). In other words, a simple working load effect (with decreased performance) or a learning effect (with improved performance) are not compatible with the present data. In addition, they are in contrast with our preliminary analysis which tested the absence of significant effect within each of the four intervals (no performance variations based on the comparison between the four time intervals for pre-feedback or for the post-feedback condition). It should also be noted that, based on correlational measures, the cortical and behavioral data showed to be matched, with a similar trend of increased values for both behavioral performance (higher RTs and ERs) and right DLPFC, as well as lower RTs and a higher left DLPFC implication.

## Conclusions

In conclusion, in the present research we observed a two-faced effect in relation to the social outcome representation when a cooperative task is performed, with significant increased right/left DLPFC activity in response to negatively or positively reinforced joint actions. This result may be explained based on the emotional significance of a positive vs. negative condition, which underlined the negative or the positive significance of a feedback in a cooperative situation (Balconi et al., 2012). At this regard, we may consider the increased right PFC responsiveness as a possible marker of the reduction of self-perceived efficacy and good performance. Indeed, as previously observed, the frontal cortical asymmetry in favor of the right hemisphere is associated with withdrawal motivations in opposition to approach motivations, where subjects have to recalibrate their strategy and to manage negative feelings linked to the inefficient performance. On the other hand, the reinforced sense of efficacy and of a better performance may induce a “positive color” on the existing relationships and the functional strategies adopted.

Some limits may be suggested for the present research. Firstly, a specific analysis should be conducted to more extensively explore the whole cortical map of cooperative behavior, considering also the contribution of posterior areas in addition to PFC. Secondly, the implementation of other alternative tasks, which may more directly represent some ecological conditions of cooperation, is to be considered for future research. Finally, a connectivity approach, able to evaluate the brain-to-brain coupling between the two inter-agents, could be suggested, to better explore the synergic inter-brain activity during cooperation in different scenarios.

## Author contributions

MB planned the experiment, supervised the data acquisition; analyzed that data, wrote the paper. MV executed the experiment, analyzed the data, and partially wrote the paper.

### Conflict of interest statement

The authors declare that the research was conducted in the absence of any commercial or financial relationships that could be construed as a potential conflict of interest.
